# Supplementary material for: Synergy of Regenerative Periodontal Surgery and Orthodontics Improves Quality of Life of Patients with Stage IV Periodontitis: 24-Month Outcomes of a Multicenter RCT
Source: Bioengineering (Basel). 2023 Jun 7;10(6):695. doi: 10.3390/bioengineering10060695 (PMC10295428; doi:10.3390/bioengineering10060695)
Supplement: Supplementary file 1 [file bioengineering-10-00695-s001.zip › bioengineering-2388209-supplementary.pdf]

Table s1. Questionaire for patient reported outcomes (impact on patients´ OHrQoL). The following 12 questions included pain and discomfort, aesthetics and functional impacts and were presented to the patients on a 5-point scale from 'never' = 0 to 'always' = 4. Patients we informed in detail about the scope of each question as follows:

In the past 3 months:

1. How often did you limit the kinds or amounts of food you eat because of problems with your teeth or dentures?
2. How often have you trouble biting or chewing any kinds of food?
3. How often were you able to swallow comfortably?
4. How often did you have problems to speak clearly?
5. How often were you able to eat anything without feeling discomfort?
6. How often did you limit contacts with people because of the condition of your teeth ?
7. How often were you pleased or happy with the looks of your teeth and gums, or dentures?
8. How often did you use medication to relieve pain?
9. How often were you worried or concerned about teeth, gums?
10. How often did you feel nervous or self-conscious/nervous because of your teeth?
11. How often did you feel uncomfortable eating in front of others?
12. How often felt teeth, gums sensitive to hot, cold or sweet foods

CONSORT 2010 checklist of information to include when reporting a randomised trial*

Section/Topic	Item No	Checklist item	Reported on page No
**Title and abstract**			
	1a	Identification as a randomised trial in the title	P 1
	1b	Structured summary of trial design, methods, results, and conclusions (for specific guidance see CONSORT for abstracts)	P 2
**Introduction**			
Background and objectives	2a	Scientific background and explanation of rationale	P 3,4
	2b	Specific objectives or hypotheses	P 4
**Methods**			
Trial design	3a	Description of trial design (such as parallel, factorial) including allocation ratio	P 4,5
	3b	Important changes to methods after trial commencement (such as eligibility criteria), with reasons	na
Participants	4a	Eligibility criteria for participants	P 5
	4b	Settings and locations where the data were collected	P 5
Interventions	5	The interventions for each group with sufficient details to allow replication, including how and when they were actually administered	P 5,6
Outcomes	6a	Completely defined pre-specified primary and secondary outcome measures, including how and when they were assessed	P 6,7
	6b	Any changes to trial outcomes after the trial commenced, with reasons	na
Sample size	7a	How sample size was determined	P 5
	7b	When applicable, explanation of any interim analyses and stopping guidelines	na
Randomisation:			
Sequence generation	8a	Method used to generate the random allocation sequence	P 5
	8b	Type of randomisation; details of any restriction (such as blocking and block size)	P 5
Allocation concealment mechanism	9	Mechanism used to implement the random allocation sequence (such as sequentially numbered containers), describing any steps taken to conceal the sequence until interventions were assigned	P 5
Implementation	10	Who generated the random allocation sequence, who enrolled participants, and who assigned participants to interventions	P 5
Blinding	11a	If done, who was blinded after assignment to interventions (for example, participants, care providers, those	P 5

		assessing outcomes) and how	
	11b	If relevant, description of the similarity of interventions	na
Statistical methods	12a	Statistical methods used to compare groups for primary and secondary outcomes	P 7
	12b	Methods for additional analyses, such as subgroup analyses and adjusted analyses	na
**Results**			
Participant flow (a diagram is strongly recommended)	13a	For each group, the numbers of participants who were randomly assigned, received intended treatment, and were analysed for the primary outcome	Figure 1
	13b	For each group, losses and exclusions after randomisation, together with reasons	Figure 1
Recruitment	14a	Dates defining the periods of recruitment and follow-up	P 5
	14b	Why the trial ended or was stopped	na
Baseline data	15	A table showing baseline demographic and clinical characteristics for each group	P 5
Numbers analysed	16	For each group, number of participants (denominator) included in each analysis and whether the analysis was by original assigned groups	Figure 1
Outcomes and estimation	17a	For each primary and secondary outcome, results for each group, and the estimated effect size and its precision (such as 95% confidence interval)	Tables 1,2,3,and Figure 2
	17b	For binary outcomes, presentation of both absolute and relative effect sizes is recommended	Tables 1, 2, 3
Ancillary analyses	18	Results of any other analyses performed, including subgroup analyses and adjusted analyses, distinguishing pre-specified from exploratory	na
Harms	19	All important harms or unintended effects in each group (for specific guidance see CONSORT for harms)	na
**Discussion**			
Limitations	20	Trial limitations, addressing sources of potential bias, imprecision, and, if relevant, multiplicity of analyses	P 14
Generalisability	21	Generalisability (external validity, applicability) of the trial findings	P 14
Interpretation	22	Interpretation consistent with results, balancing benefits and harms, and considering other relevant evidence	P 11-14
**Other information**			
Registration	23	Registration number and name of trial registry	P 4
Protocol	24	Where the full trial protocol can be accessed, if available	P 4
Funding	25	Sources of funding and other support (such as supply of drugs), role of funders	P 16

*We strongly recommend reading this statement in conjunction with the CONSORT 2010 Explanation and Elaboration for important clarifications on all the items. If relevant, we also recommend reading CONSORT extensions for cluster randomised trials, non-inferiority and equivalence trials, non-pharmacological treatments, herbal interventions, and pragmatic trials. Additional extensions are forthcoming: for those and for up to date references relevant to this checklist, see www.consort-statement.org.
